# Supplementary material for: Response diversity of free-floating plants to nutrient stoichiometry and temperature: growth and resting body formation
Source: PeerJ. 2016 Mar 7;4:e1781. doi: 10.7717/peerj.1781 (PMC4793347; doi:10.7717/peerj.1781)
Supplement: Supplemental Information 3 [file peerj-04-1781-s003.docx]

**Title:** Response diversity of free-floating plants to nutrient stoichiometry and temperature: Growth and resting body formation

**Author:** Michael J. McCann ^a, 1^

^a^ **Affiliation:** Department of Ecology and Evolution, Stony Brook University, 650 Life Sciences Building, Stony Brook, New York 11794-5245 USA

^1^ **Present address:** Department of Marine and Coastal Sciences, Rutgers University, 71 Dudley Road, New Brunswick, New Jersey 08901-8525 USA

**E-mail:** mccannmikejames@gmail.com

**Appendix A.** Converting from turion number to turion area

Since turion size differs between species, turion area, rather than number, is a better means of comparison among species. Turion number, but not area, was recorded in the experiments described in the main text. Therefore, I used data from a separate experiment where both responses were measured to develop a conversion between turion number and turion area.

I grew *Spirodela polyrhiza* and *Wolffia brasiliensis* under conditions similar to the second experiment described in the main text (Experiment II, nutrient stoichiometry). Plants were grown for 12 day and nutrient media were changed on days 3, 5, 7, and 10. Each time nutrient media were changed, I counted the number of turions present in each replicate. I also photographed the turions, while still in the multiwell plates. I measured their area following the methods described in the main text.

I fit a least square regression between turion number and turion area for *W. brasiliensis* with robust standard errors to account for heteroskedascity (Figure A1). I used the slope (0.184) and intercept (-0.005) from this regression line to estimate the area of turions in the experiments when only the number of turions was recorded. On the other hand, *S. polyrhiza* typically formed a single turion between each nutrient medium changes. Therefore, I used the mean turion area (2.79 mm^2^) to estimate the area of *S. polyrhiza* turions in the experiments where only the number of turions was recorded (Figure A2).


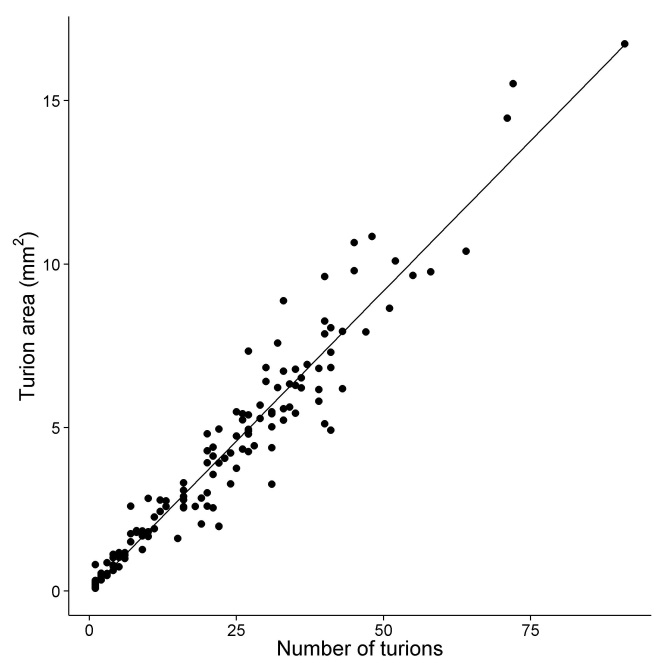


**Figure A1.**The relationship between turion number and turion area of *Wolffia brasiliensis* (n = 140, least squares regression with robust standard errors to account for heteroskedascity, turion area = 0.184 * turion number – 0.005, p < 0.001, adjusted R^2^ = 0.926).


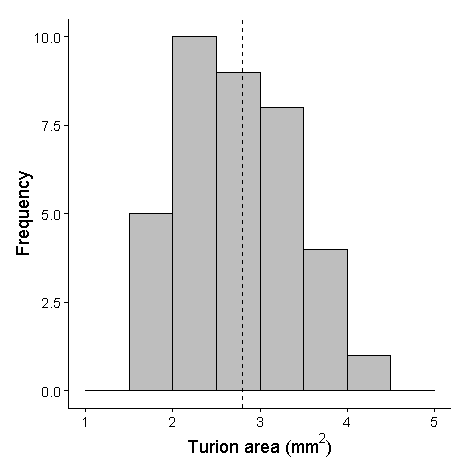


**Figure A2.**The frequency distribution of area of single turions of *Spirodela polyrhiza* (n = 37)*.* Dashed line indicates the average value (2.79 mm^2^).
