# Supplementary material for: Response diversity of free-floating plants to nutrient stoichiometry and temperature: growth and resting body formation
Source: PeerJ. 2016 Mar 7;4:e1781. doi: 10.7717/peerj.1781 (PMC4793347; doi:10.7717/peerj.1781)
Supplement: Supplemental Information 4 [file peerj-04-1781-s004.docx]

**Title:** Response diversity of free-floating plants to nutrient stoichiometry and temperature: Growth and resting body formation

**Author:** Michael J. McCann ^a, 1^

^a^ **Affiliation:** Department of Ecology and Evolution, Stony Brook University, 650 Life Sciences Building, Stony Brook, New York 11794-5245 USA

^1^ **Present address:** Department of Marine and Coastal Sciences, Rutgers University, 71 Dudley Road, New Brunswick, New Jersey 08901-8525 USA

**E-mail:** mccannmikejames@gmail.com

**Appendix B.** Analysis of growth rates using factorial ANOVAs.

An alternative approach to analyzing the growth rate data from both experiments is two perform three-way factorial ANOVAs to test for the main and interactive effects of species and experimental conditions on growth rates. Data were tested for normally distributed residuals with a Shapiro-Wilk test and equal variance among treatment groups with Levene’s test. If the data did not meet these assumptions of ANOVA, they were power transformed to ensure that these criteria were met. I performed all statistical analyses in R version 3.0.2 (R Development Core Team 2013).

**Table B1.** Three-way ANOVA table for the effect of species, nutrients, and temperature on average growth rate (Experiment I).

| Term | df | SS | MS | F | p |
| --- | --- | --- | --- | --- | --- |
| Species | 2 | 0.0787 | 0.0393 | 82.612 | <0.001 |
| Nutrients | 2 | 0.7094 | 0.3547 | 744.73 | <0.001 |
| Temperature | 2 | 0.0146 | 0.0073 | 15.35 | <0.001 |
| Species * Nutr. | 4 | 0.0134 | 0.0034 | 7.047 | <0.001 |
| Species * Temp. | 4 | 0.0145 | 0.0036 | 7.622 | <0.001 |
| Nutr. * Temp. | 4 | 0.0128 | 0.0032 | 6.693 | <0.001 |
| Species * Nutr. * Temp. | 8 | 0.004 | 0.0005 | 1.043 | 0.405 |
| Residuals | 188 | 0.0895 | 0.0005 |  |  |

**Table B2.** Three-way ANOVA table for the effect of species, nitrogen, and phosphorus on average growth rate (log x + 1 transformed) (Experiment II).

| Term | df | SS | MS | F | p |
| --- | --- | --- | --- | --- | --- |
| Species | 2 | 0.0999 | 0.04994 | 35.569 | <0.001 |
| Nitrogen | 2 | 0.0385 | 0.01927 | 13.724 | <0.001 |
| Phosphorus | 2 | 0.1784 | 0.0892 | 63.534 | <0.001 |
| Species * Nitrogen | 4 | 0.0268 | 0.0067 | 4.773 | 0.001 |
| Species * Phosphorus | 4 | 0.0081 | 0.00203 | 1.445 | 0.223 |
| Nitrogen * Phosphorus | 4 | 0.0494 | 0.01235 | 8.795 | <0.001 |
| Species * N * P | 8 | 0.011 | 0.00138 | 0.982 | 0.453 |
| Residuals | 135 | 0.1895 | 0.0014 |  |  |
