# Supplementary material for: Response diversity of free-floating plants to nutrient stoichiometry and temperature: growth and resting body formation
Source: PeerJ. 2016 Mar 7;4:e1781. doi: 10.7717/peerj.1781 (PMC4793347; doi:10.7717/peerj.1781)
Supplement: Supplemental Information 5 [file peerj-04-1781-s005.docx]

**Title:** Response diversity of free-floating plants to nutrient stoichiometry and temperature: Growth and resting body formation

**Author:** Michael J. McCann ^a, 1^

^a^ **Affiliation:** Department of Ecology and Evolution, Stony Brook University, 650 Life Sciences Building, Stony Brook, New York 11794-5245 USA

^1^ **Present address:** Department of Marine and Coastal Sciences, Rutgers University, 71 Dudley Road, New Brunswick, New Jersey 08901-8525 USA

**E-mail:** mccannmikejames@gmail.com

**Appendix C.** Properties of 205 surveyed water bodies.

Note: Phosphorus, conductivity, and Secchi depth are average values. FP = floating plant. A = *Azolla* sp., **LM** = *Lemna minor*, LT = *Lemna trisulca*, LV = *Lemna valdiviana* , R = *Riccia* sp., **SP** = *Spirodela polyrhiza*, **W** = *Wolffia* sp. Species in bold were included in the lab experiments in this study.

| FP richness | FP composition | FP cover (%) | Latitude | Longitude | Size (ha) | Depth, max (m) | Total phosphorus (ug/L) | Conductivity (us/cm) | Secchi depth (m) |
| --- | --- | --- | --- | --- | --- | --- | --- | --- | --- |
| 4 | **LM**, R, **SP**, **W** | 44.6 | 40.85736 | 73.21032 | 0.1 | 1.0 | 17.68 | 239.3 |  |
| 4 | A, **LM, SP, W** | 100.0 | 41.17 | 73.35631 | 0.4 | 2.1 | 118.6 | 174.0 | 2.1 |
| 4 | **LM,** LV, **SP, W** | 0.4 | 40.76994 | 72.99316 | 4.39 | 2.1 | 22.2 | 175.6 |  |
| 3 | **LM, SP, W** | 81.3 | 40.88422 | 73.36119 | 0.59 | 1.5 | 19.72 | 218.2 |  |
| 3 | **LM, SP, W** | 5.0 | 40.84021 | 73.22489 | 40.67 | 2.4 | 20.95 | 178.0 |  |
| 3 | **LM, SP, W** | 0.2 | 41.78829 | 72.45363 | 8.5 | 1.5 | 13.5 | 413.5 | 1.0 |
| 3 | **LM, SP, W** | 2.8 | 41.29532 | 72.80171 | 7.04 | 4.0 | 81.5 | 236.3 | 1.5 |
| 3 | **LM, SP, W** | 0.1 | 41.84679 | 71.83803 | 28.77 | 3.0 | 21.5 | 67.5 | 2.7 |
| 3 | **LM, SP, W** | 0.0 | 41.56392 | 71.87308 | 27.68 | 2.0 | 51.5 | 58.1 | 1.0 |
| 3 | LT, SP, W | 17.3 | 41.68358 | 73.47777 | 26.71 | 4.0 | 157.4 | 257.0 | 2.0 |
| 3 | **LM, SP, W** | 100.0 | 41.31595 | 72.70061 | 0.0858 | 1.0 | 28.15 | 64.0 |  |
| 3 | **LM**, LT, SP | 2.4 | 41.18232 | 73.42648 | 2.23 | 1.0 | 54.05 | 394.5 | 0.8 |
| 3 | **LM, SP, W** | 16.7 | 40.83468 | 72.91814 | 10.12 | 2.1 | 18.4 | 154.3 |  |
| 3 | **LM, SP, W** | 14.2 | 41.44279 | 72.77219 | 59.8 | 3.0 | 38.5 | 248.9 | 0.9 |
| 3 | **LM, SP, W** | 56.5 | 40.94646 | 73.11558 | 1.5 | 2.3 | 25.79 | 210.6 |  |
| 3 | **LM, SP, W** | 69.7 | 40.94435 | 73.11602 | 1.13 | 1.2 | 23.66 | 209.0 |  |
| 3 | **LM, SP, W** | 100.0 | 41.82456 | 72.91158 | 0.4 | 1.5 | 40.2 | 238.0 | 1.0 |
| 3 | **LM, SP, W** | 5.0 | 41.48544 | 72.77947 | 26.06 | 2.0 | 111.6 | 176.3 | 0.8 |
| 3 | **LM**, LT, SP | 0.3 | 41.39442 | 72.69666 | 39.94 | 14.0 | 10.1 | 116.6 | 3.5 |
| 3 | **LM, SP, W** | 1.0 | 40.90216 | 72.79315 | 23.22 | 1.8 | 19.55 | 78.6 |  |
| 3 | **LM, SP, W** | 100.0 | 41.07807 | 73.47213 | 0.85 | 0.8 | 183.6 | 239.5 | 0.8 |
| 3 | **LM, SP, W** | 4.7 | 41.29348 | 72.69152 | 1.17 | 2.0 | 37.4 | 188.2 | 2.0 |
| 3 | **LM, SP, W** | 100.0 | 41.2301 | 73.41338 | 0.16 | 1.5 | 178 | 152.5 | 0.7 |
| 3 | **LM, SP, W** | 100.0 | 41.27087 | 72.77647 | 1.7 | 1.0 | 68.3 | 199.8 | 0.3 |
| 2 | **LM, W** | 100.0 | 40.96239 | 73.12848 | 0.4 | 1.0 | 386.64 | 116.4 |  |
| 2 | **LM, SP** | 26.2 | 41.69298 | 72.53177 | 3.8 | 1.2 | 39 | 126.5 | 1.2 |
| 2 | **LM, SP** | 0.2 | 41.56859 | 72.78691 | 12.23 | 3.0 | 47.6 | 318.5 | 2.0 |
| 2 | **LM, SP** | 0.1 | 41.92419 | 73.43413 | 10.93 | 4.7 |  |  | 1.3 |
| 2 | **LM, W** | 0.0 | 41.51539 | 72.73139 | 45.73 | 7.3 | 32 | 125.8 | 2.8 |
| 2 | **LM, SP** | 91.1 | 41.09058 | 73.46373 | 0.27 | 1.0 | 183.71 | 236.4 | 0.4 |
| 2 | **LM, W** | 30.0 | 41.50755 | 72.76606 | 4.7394 | 1.5 | 30.04 | 496.0 |  |
| 2 | **LM, W** | 0.1 | 41.56374 | 72.03777 | 11.9 | 1.7 | 57 | 104.5 | 1.7 |
| 2 | **LM, SP** | 0.3 | 41.37191 | 72.122 | 11.94 | 3.0 | 74 | 159.0 | 1.5 |
| 2 | **LM, SP** | 1.0 | 41.93605 | 72.37389 | 75.84 | 12.0 | 55 | 147.0 | 2.9 |
| 2 | **LM, SP** | 2.7 | 41.40869 | 72.80267 | 2.67 | 2.0 | 39.6 | 118.0 | 1.4 |
| 2 | **LM, W** | 24.8 | 41.07916 | 73.46074 | 2.99 | 4.0 | 97.5 | 487.5 | 1.4 |
| 2 | **LM, SP** | 0.2 | 41.52323 | 73.14099 | 6.27 | 2.0 | 55 | 173.0 | 1.2 |
| 2 | **LM, W** | 0.6 | 41.67057 | 72.8475 | 4.86 | 4.0 | 89.5 | 262.5 | 1.2 |
| 2 | **LM, W** | 100.0 | 41.35059 | 72.8517 | 0.5766 | 1.0 | 63.96 | 329.0 |  |
| 2 | **LM, W** | 3.3 | 41.25053 | 73.00999 | 10.93 | 4.0 | 69 | 228.5 | 1.0 |
| 2 | **LM, W** | 16.1 | 41.80343 | 72.77213 | 1.09 | 1.0 | 53 | 178.0 | 1.3 |
| 2 | **LM, W** | 20.6 | 41.46451 | 72.85655 | 0.4 | 2.5 | 122.3 | 184.1 | 0.7 |
| 2 | **LM, W** | 0.9 | 41.44279 | 72.77219 | 135.34 | 3.8 | 31.7 | 210.8 |  |
| 2 | **LM, SP** | 49.4 | 41.69472 | 72.73002 | 1.46 | 2.8 | 94.8 | 444.1 | 1.2 |
| 2 | **LM, SP** | 24.0 | 41.82656 | 72.91411 | 1.78 | 1.5 | 67.1 | 224.4 | 1.0 |
| 2 | **LM, SP** | 80.0 | 41.3165 | 72.7223 | 0.0275 | 1.0 | 22.34 | 153.0 |  |
| 2 | A, **W** | 100.0 | 42.00276 | 72.6463 | 0.12 | 0.5 | 203.5 | 184.0 | 0.5 |
| 2 | **SP, W** | 0.2 | 41.53184 | 73.15327 | 119.79 | 10.0 |  | 67.7 | 4.0 |
| 2 | **LM, SP** | 1.7 | 41.34442 | 73.05247 | 0.49 | 1.0 | 18 | 58.0 | 1.1 |
| 2 | **LM, SP** | 2.9 | 41.13459 | 73.42225 | 0.4 | 2.0 | 168.8 | 206.0 | 0.6 |
| 2 | **SP, W** | 10.9 | 41.5794 | 72.77293 | 59.08 | 4.9 | 72.8 | 247.3 | 1.4 |
| 2 | **LM, W** | 5.4 | 41.60271 | 72.86369 | 3.08 | 1.3 | 17 | 263.0 | 1.3 |
| 2 | **LM, W** | 0.2 | 41.504 | 72.407 | 178.06 | 3.0 | 69.7 | 55.0 | 1.5 |
| 2 | **LM, SP** | 0.3 | 40.84342 | 72.93702 | 7.4 | 1.8 | 18.29 | 153.0 |  |
| 1 | **LM** | 0.1 | 41.44757 | 72.99517 | 2.19 | 3.5 | 19.5 | 129.5 | 2.0 |
| 1 | **LM** | 0.5 | 41.46259 | 73.52383 | 33.39 | 15.5 | 184.5 | 262.5 | 2.0 |
| 1 | **LM** | 1.1 | 41.20738 | 73.18788 | 13.52 | 5.0 | 17.5 | 159.0 | 2.0 |
| 1 | **LM** | 0.0 | 41.25132 | 73.239 | 25.78 | 6.0 | 50.7 | 153.0 | 3.1 |
| 1 | **LM** | 0.1 | 41.32061 | 72.77994 | 8.82 | 5.3 | 24.5 | 284.5 | 2.3 |
| 1 | **LM** | 0.0 | 42.02516 | 72.52218 | 14.16 | 2.5 | 164 | 152.5 | 1.0 |
| 1 | **LM** | 9.3 | 41.30111 | 72.41396 | 2.23 | 1.2 | 42.5 | 101.2 | 0.6 |
| 1 | **LM** | 6.4 | 41.64631 | 72.63289 | 0.53 | 1.0 | 52.1 | 355.5 | 1.0 |
| 1 | **LM** | 2.9 | 41.64575 | 72.63426 | 2.35 | 1.0 | 173.3 | 331.0 | 0.9 |
| 1 | **LM** | 0.8 | 41.60138 | 73.09778 | 4.33 | 2.0 | 48 | 193.5 | 1.2 |
| 1 | **LM** | 0.2 | 41.39612 | 72.9421 | 6.07 | 2.0 | 164.7 | 51.0 | 0.3 |
| 1 | **LM** | 1.0 | 40.84475 | 73.13923 | 1.61 | 2.5 | 36.98 | 285.3 | 0.5 |
| 1 | **LM** | 0.2 | 41.30494 | 72.85553 | 7.89 | 3.0 | 18.7 | 209.7 | 4.0 |
| 1 | **SP** | 0.1 | 41.84019 | 72.10908 | 33.18 | 4.3 | 7 | 44.1 | 3.0 |
| 1 | **W** | 44.0 | 41.28351 | 72.58265 | 3.08 | 2.0 | 75.5 | 196.5 | 1.5 |
| 1 | **SP** | 0.1 | 41.91828 | 73.49461 | 82.96 | 3.0 | 14 | 116.3 | 2.0 |
| 1 | **LM** | 0.9 | 41.50103 | 73.11168 | 3.44 | 3.0 | 56 | 130.0 | 0.8 |
| 1 | **LM** | 0.0 | 41.51451 | 72.42203 | 50.99 | 4.0 | 34 | 72.3 | 1.8 |
| 1 | **LM** | 0.1 | 41.32008 | 73.52723 | 35.7 | 3.4 | 52.4 | 271.3 | 1.4 |
| 1 | **LM** | 100.0 | 40.89723 | 73.14835 | 0.36 | 1.0 | 88.62 | 1120.3 | 0.5 |
| 1 | **LM** | 0.0 | 41.41707 | 73.13694 | 5.14 | 1.5 | 52.5 | 77.7 | 0.9 |
| 1 | **LM** | 0.8 | 41.33061 | 72.73172 | 0.2 | 1.8 | 91 | 148.5 | 1.0 |
| 1 | **W** | 66.1 | 41.2947 | 72.62439 | 0.4 | 3.0 | 42.5 | 294.0 | 1.8 |
| 1 | **SP** | 1.8 | 41.3741 | 72.23028 | 51.8 | 10.1 | 7.8 | 69.5 | 2.5 |
| 1 | A | 100.0 | 42.00268 | 72.64729 | 0.28 | 0.5 | 1424.6 | 336.0 | 0.5 |
| 1 | LT | 0.1 | 41.3388 | 73.37758 | 5.67 | 2.5 | 20 | 106.5 | 1.5 |
| 1 | **LM** | 90.0 | 40.76478 | 72.98207 | 3.34 | 0.5 | 35.23 | 222.4 |  |
| 1 | **SP** | 0.0 | 41.95089 | 71.95093 | 38.85 | 5.0 | 208.5 | 95.0 | 1.5 |
| 1 | **LM** | 3.2 | 41.71794 | 72.60965 | 0.45 | 2.0 | 99.5 | 116.0 | 0.7 |
| 1 | **LM** | 71.1 | 41.10905 | 73.44343 | 0.97 | 2.0 | 504.5 | 117.5 | 1.5 |
| 1 | **LM** | 0.0 | 41.31925 | 73.11065 | 2.95 | 3.0 | 41.5 | 153.0 | 2.2 |
| 1 | **LM** | 0.4 | 41.59107 | 72.83903 | 5.99 | 3.0 |  |  | 2.1 |
| 1 | **W** | 100.0 | 40.96768 | 72.62792 | 0.24 | 1.0 | 457.17 | 55.0 |  |
| 1 | **LM** | 5.0 | 41.26027 | 72.74879 | 0.0309 | 1.0 | 206.42 | 983.0 |  |
| 1 | **LM** | 8.0 | 41.26764 | 72.78897 | 0.45 | 1.0 | 107 | 204.5 | 1.0 |
| 1 | **LM** | 0.5 | 41.26131 | 72.83749 | 1.21 | 1.0 | 65 | 52.0 |  |
| 1 | **SP** | 0.6 | 41.41 | 73.33179 | 50.18 | 10.0 | 148.5 | 147.5 | 2.0 |
| 1 | **SP** | 10.1 | 41.82003 | 72.82271 | 5.42 | 2.0 | 42.1 | 250.8 | 1.5 |
| 1 | **SP** | 4.0 | 41.81764 | 72.82342 | 6.6 | 3.5 | 21.8 | 313.5 | 2.0 |
| 1 | **W** | 100.0 | 41.31642 | 72.78173 | 0.1743 | 1.0 | 28.77 | 252.0 |  |
| 1 | **LM** | 0.0 | 41.44672 | 72.74408 | 62.32 | 7.0 | 28.5 | 136.0 | 2.6 |
| 1 | **LM** | 1.5 | 41.41692 | 72.8846 | 0.57 | 2.0 | 28 | 132.0 | 2.0 |
| 1 | **LM** | 0.1 | 41.34991 | 73.45925 | 14.45 | 2.5 | 13 | 199.0 | 2.4 |
| 1 | **LM** | 100.0 | 41.33397 | 72.7438 | 0.0281 | 1.0 | 78.24 | 156.0 |  |
| 1 | **LM** | 0.1 | 41.43822 | 71.96957 | 3.2 | 2.0 | 74 | 97.5 | 1.5 |
| 0 |  | 0.0 | 41.5904 | 72.42655 | 0.36 | 3.0 | 16.5 | 91.7 | 1.5 |
| 0 |  | 0.0 | 41.86072 | 71.89911 | 87.01 | 15.5 | 28 | 30.3 | 5.8 |
| 0 |  | 0.0 | 41.51468 | 71.97423 | 45.73 | 14.0 | 22.2 | 116.5 | 1.7 |
| 0 |  | 0.0 | 41.62651 | 72.33073 | 75.68 | 7.9 | 53 | 67.9 | 3.5 |
| 0 |  | 0.0 | 41.50827 | 71.89536 | 22.91 | 2.3 | 50.7 | 69.3 | 1.4 |
| 0 |  | 0.0 | 41.72207 | 72.3592 | 63.54 | 3.0 | 13 | 76.7 | 2.0 |
| 0 |  | 0.0 | 41.9005 | 72.12765 | 21.45 | 3.7 | 7.5 | 93.0 | 1.1 |
| 0 |  | 0.0 | 41.49308 | 71.97971 | 10.4 | 2.0 | 84 | 114.0 | 1.2 |
| 0 |  | 0.0 | 41.79429 | 73.19495 | 10 | 2.0 | 56.5 | 144.0 | 1.4 |
| 0 |  | 0.0 | 41.28515 | 72.76449 | 0.04 | 3.0 | 29.5 | 223.5 | 1.0 |
| 0 |  | 0.0 | 41.57596 | 71.78904 | 150.54 | 14.5 | 12.5 | 42.4 | 5.0 |
| 0 |  | 0.0 | 41.25058 | 73.14075 | 23.39 | 12.0 | 16.5 | 245.0 | 2.0 |
| 0 |  | 0.0 | 41.99266 | 72.13085 | 10.12 | 4.7 | 36.5 | 67.9 | 2.5 |
| 0 |  | 0.0 | 41.50757 | 71.87318 | 39.42 | 10.1 | 3.5 | 38.0 | 5.4 |
| 0 |  | 0.0 | 41.9231 | 72.21908 | 19.63 | 2.0 | 33 | 80.6 | 1.0 |
| 0 |  | 0.0 | 41.34555 | 72.29826 | 5.1 | 4.9 | 59 | 127.5 | 2.8 |
| 0 |  | 0.0 | 41.96955 | 72.06934 | 29.7 | 7.0 | 16.5 | 75.9 | 3.8 |
| 0 |  | 0.0 | 41.80132 | 72.433 | 70.82 | 6.1 | 13.3 | 103.9 | 3.1 |
| 0 |  | 0.0 | 41.8089 | 72.42619 | 48.97 | 6.1 | 29.2 | 95.2 | 2.9 |
| 0 |  | 0.0 | 41.82184 | 72.42102 | 20.23 | 2.3 | 30 | 62.1 | 0.8 |
| 0 |  | 0.0 | 41.91115 | 72.53823 | 0.81 | 2.0 | 36.6 | 236.0 | 2.0 |
| 0 |  | 0.0 | 41.91006 | 72.53856 | 0.32 | 0.5 | 40.7 | 264.0 | 0.5 |
| 0 |  | 0.0 | 41.87184 | 73.09576 | 34.44 | 3.4 | 6 | 93.2 | 2.4 |
| 0 |  | 0.0 | 41.30587 | 72.52501 | 3.44 | 11.0 | 18.3 | 79.5 | 1.4 |
| 0 |  | 0.0 | 41.40168 | 72.49901 | 28.04 | 13.0 | 25.1 | 37.1 | 2.9 |
| 0 |  | 0.0 | 41.93943 | 72.19431 | 21.04 | 3.3 | 18.5 | 130.5 | 2.3 |
| 0 |  | 0.0 | 41.30229 | 72.39669 | 4.17 | 1.0 | 33 | 146.0 | 0.5 |
| 0 |  | 0.0 | 41.33719 | 72.74107 | 8.09 | 2.7 | 59 | 199.5 | 3.0 |
| 0 |  | 0.0 | 41.77093 | 72.31974 | 150.95 | 10.0 | 34.5 | 57.5 | 3.3 |
| 0 |  | 0.0 | 41.52188 | 72.63876 | 12.55 | 6.0 | 37.5 | 121.1 | 1.5 |
| 0 |  | 0.0 | 41.92278 | 72.09932 | 58.68 | 5.0 | 80.5 | 63.6 | 2.3 |
| 0 |  | 0.0 | 41.38917 | 73.03082 | 0.08 | 2.0 | 18.5 | 82.5 | 2.0 |
| 0 |  | 0.0 | 41.34356 | 72.58872 | 12.95 | 2.3 | 17 | 65.6 | 1.3 |
| 0 |  | 0.0 | 41.34116 | 72.59215 | 2.43 | 5.0 | 17 | 65.6 | 3.7 |
| 0 |  | 0.0 | 41.69494 | 72.48231 | 8.09 | 4.0 | 11 | 67.5 | 2.5 |
| 0 |  | 0.0 | 41.81353 | 73.2388 | 26.63 | 3.4 | 43 | 145.0 | 2.5 |
| 0 |  | 0.0 | 41.78611 | 72.28203 | 32.21 | 2.5 |  | 137.5 | 2.1 |
| 0 |  | 0.0 | 41.64861 | 72.98933 | 12.14 | 1.7 | 12 | 183.0 | 1.7 |
| 0 |  | 0.0 | 41.32004 | 72.67371 | 6.84 | 5.0 | 29.5 | 75.1 | 2.3 |
| 0 |  | 0.0 | 41.21996 | 73.20664 | 26.71 | 6.0 | 16 | 143.0 | 1.8 |
| 0 |  | 0.0 | 41.74178 | 72.84136 | 5.58 | 3.0 | 14 | 183.0 | 2.6 |
| 0 |  | 0.0 | 41.50874 | 72.22279 | 214.08 | 11.9 | 11 | 79.0 | 2.0 |
| 0 |  | 0.0 | 41.39102 | 72.5576 | 0.32 | 2.5 | 69.5 | 76.0 | 0.8 |
| 0 |  | 0.0 | 41.57392 | 72.56294 | 30.76 | 2.7 | 2 | 52.0 | 2.4 |
| 0 |  | 0.0 | 41.53006 | 71.80907 | 19.51 | 7.9 | 3 | 20.1 | 5.6 |
| 0 |  | 0.0 | 41.51878 | 72.32729 | 69.61 | 10.7 | 13.8 | 103.5 | 2.4 |
| 0 |  | 0.0 | 41.18087 | 73.37183 | 12.71 | 13.0 | 16.5 | 205.0 | 2.5 |
| 0 |  | 0.0 | 41.42103 | 72.57104 | 15.78 | 2.0 | 18.5 | 75.8 | 1.9 |
| 0 |  | 0.0 | 41.54686 | 72.72233 | 24.73 | 6.0 | 14.5 | 102.5 | 1.0 |
| 0 |  | 0.0 | 41.54399 | 72.72083 | 7.28 | 2.0 | 41.5 | 156.0 | 0.8 |
| 0 |  | 0.0 | 41.53777 | 72.72257 | 17.12 | 1.8 | 44 | 154.0 | 1.5 |
| 0 |  | 0.0 | 41.55274 | 72.72902 | 11.29 | 4.0 | 4 | 44.0 | 1.0 |
| 0 |  | 0.0 | 41.46175 | 72.25084 | 5.34 | 5.1 | 30 | 81.0 | 2.6 |
| 0 |  | 0.0 | 41.54433 | 72.60446 | 2.39 | 5.5 | 6 | 52.3 | 4.0 |
| 0 |  | 0.0 | 41.32818 | 73.10852 | 132.74 | 7.9 | 20 | 238.0 | 2.0 |
| 0 |  | 0.0 | 42.02222 | 72.99288 | 5.79 | 2.5 | 37.5 | 29.5 | 1.2 |
| 0 |  | 0.0 | 41.29901 | 72.39684 | 5.79 | 10.0 | 39 | 78.3 | 3.7 |
| 0 |  | 0.0 | 41.34751 | 72.43418 | 1.01 | 2.0 | 55 | 89.5 | 2.0 |
| 0 |  | 0.0 | 41.36054 | 72.70377 | 4.45 | 1.0 | 38 | 112.5 | 0.8 |
| 0 |  | 0.0 | 41.88743 | 73.01997 | 6.47 | 7.0 | 54.5 | 111.5 | 3.6 |
| 0 |  | 0.0 | 41.31704 | 72.78478 | 9.27 | 13.4 | 174 | 299.0 | 4.5 |
| 0 |  | 0.0 | 41.6535 | 73.20825 | 40.47 | 2.1 | 96.4 | 88.1 | 1.9 |
| 0 |  | 0.0 | 41.39284 | 72.37909 | 1.62 | 1.7 | 16.5 | 54.8 | 1.6 |
| 0 |  | 0.0 | 41.63474 | 73.11386 | 14.37 | 3.0 | 39.5 | 100.5 | 1.5 |
| 0 |  | 0.0 | 41.33882 | 72.48377 | 29.54 | 4.9 | 35.5 | 130.0 | 2.8 |
| 0 |  | 0.0 | 41.47727 | 72.63283 | 13.19 | 5.5 | 6 | 24.0 | 4.0 |
| 0 |  | 0.0 | 41.81138 | 73.29292 | 6.56 | 8.0 | 18 | 35.9 | 5.4 |
| 0 |  | 0.0 | 41.67663 | 72.31115 | 41.28 | 2.0 | 13.2 | 23.1 | 2.0 |
| 0 |  | 0.0 | 41.73433 | 71.86675 | 38.77 | 6.0 | 108.9 | 53.1 | 3.0 |
| 0 |  | 0.0 | 41.96006 | 72.1922 | 18.13 | 3.5 | 15.5 | 227.5 | 3.5 |
| 0 |  | 0.0 | 42.02185 | 72.03045 | 15.41 | 3.0 | 10.9 | 84.0 | 2.5 |
| 0 |  | 0.0 | 41.36302 | 71.96287 | 0.12 | 2.0 | 24 | 123.5 | 1.5 |
| 0 |  | 0.0 | 41.35674 | 72.62813 | 3.28 | 1.3 | 44 | 154.0 | 1.3 |
| 0 |  | 0.0 | 41.68265 | 72.84798 | 6.39 | 2.0 | 43 | 144.0 | 0.9 |
| 0 |  | 0.0 | 41.25557 | 73.17577 | 24.36 | 4.2 | 14.8 | 121.3 | 1.5 |
| 0 |  | 0.0 | 41.43236 | 72.74155 | 55.2 | 12.0 | 15.5 | 152.5 | 3.2 |
| 0 |  | 0.0 | 41.59784 | 72.49581 | 206.79 | 7.0 | 61.5 | 107.5 | 0.5 |
| 0 |  | 0.0 | 41.39259 | 72.24905 | 58.27 | 14.0 | 16 | 23.2 | 3.0 |
| 0 |  | 0.0 | 41.38053 | 71.8605 | 1.01 | 3.0 | 9 | 79.6 | 3.0 |
| 0 |  | 0.0 | 41.37927 | 71.86087 | 0.2 | 3.0 | 29.5 | 113.9 | 1.0 |
| 0 |  | 0.0 | 41.42209 | 72.33256 | 0.01 | 0.8 | 10.2 | 29.0 | 0.8 |
| 0 |  | 0.0 | 41.33536 | 72.52367 | 0.12 | 0.5 | 93.4 | 35.0 | 1.0 |
| 0 |  | 0.0 | 41.95974 | 71.81632 | 165.11 | 6.1 | 6.5 | 52.1 | 2.8 |
| 0 |  | 0.0 | 41.36418 | 72.30096 | 105.22 | 19.2 | 6.5 | 47.0 | 2.3 |
| 0 |  | 0.0 | 41.17892 | 73.41998 | 3.56 | 5.0 | 12.3 | 291.0 | 2.0 |
| 0 |  | 0.0 | 41.3693 | 72.58971 | 1.58 | 3.0 | 43 | 66.4 | 1.7 |
| 0 |  | 0.0 | 41.30803 | 73.11969 | 4.17 | 3.4 | 28 | 102.0 | 3.0 |
| 0 |  | 0.0 | 41.36044 | 71.90922 | 8.01 | 3.0 | 90.5 | 226.0 | 1.1 |
| 0 |  | 0.0 | 41.77849 | 72.82611 | 15.05 | 2.0 | 49 | 177.5 | 0.8 |
| 0 |  | 0.0 | 41.51512 | 73.47843 | 107.65 | 12.0 | 6.5 | 119.5 | 3.5 |
| 0 |  | 0.0 | 42.01228 | 72.25539 | 60.3 | 3.5 | 22.7 | 65.8 | 2.8 |
| 0 |  | 0.0 | 41.59868 | 73.07024 | 3.68 | 2.0 | 24 | 138.0 | 1.4 |
| 0 |  | 0.0 | 41.53585 | 73.51186 | 4.86 | 3.0 | 21 | 68.5 | 2.7 |
| 0 |  | 0.0 | 41.83768 | 73.26142 | 75.68 | 7.0 | 103.3 | 114.6 | 2.3 |
| 0 |  | 0.0 | 41.33809 | 72.6815 | 5.26 | 2.0 | 27 | 66.5 | 0.6 |
| 0 |  | 0.0 | 41.87794 | 73.04411 | 99.15 | 13.0 | 10.8 | 54.5 | 8.4 |
| 0 |  | 0.0 | 41.33899 | 72.7341 | 21.12 | 5.0 | 50.2 | 236.0 | 2.5 |
| 0 |  | 0.0 | 41.85519 | 73.2565 | 16.96 | 9.0 | 215.1 | 134.3 | 4.0 |
| 0 |  | 0.0 | 41.63494 | 72.3087 | 101.17 | 2.3 | 13.5 | 56.0 | 2.3 |
| 0 |  | 0.0 | 41.70309 | 72.25823 | 0.04 | 1.5 |  | 88.5 | 1.8 |
| 0 |  | 0.0 | 41.90873 | 73.15069 | 99.55 | 5.2 | 3.5 | 34.0 | 3.0 |
| 0 |  | 0.0 | 41.58372 | 73.12765 | 45.69 | 5.0 | 30.5 | 228.0 | 1.1 |
| 0 |  | 0.0 | 41.35391 | 72.96891 | 17.97 | 8.0 | 33.1 | 46.3 | 3.1 |
| 0 |  | 0.0 | 41.73685 | 72.76538 | 24.28 | 2.0 | 36.1 | 413.5 | 2.0 |
| 0 |  | 0.0 | 41.49105 | 71.87221 | 40.8 | 8.0 | 35.8 | 56.0 | 2.2 |
| 0 |  | 0.0 | 41.19675 | 73.35435 | 0.04 | 1.0 |  |  |  |
